# Supplementary material for: Chromatin accessibility complex subunit 1 enhances tumor growth by regulating the oncogenic transcription of YAP in breast and cervical cancer
Source: PeerJ. 2024 Jan 10;12:e16752. doi: 10.7717/peerj.16752 (PMC10787542; doi:10.7717/peerj.16752)
Supplement: Supplemental Information 1 [file peerj-12-16752-s001.docx]

# 1
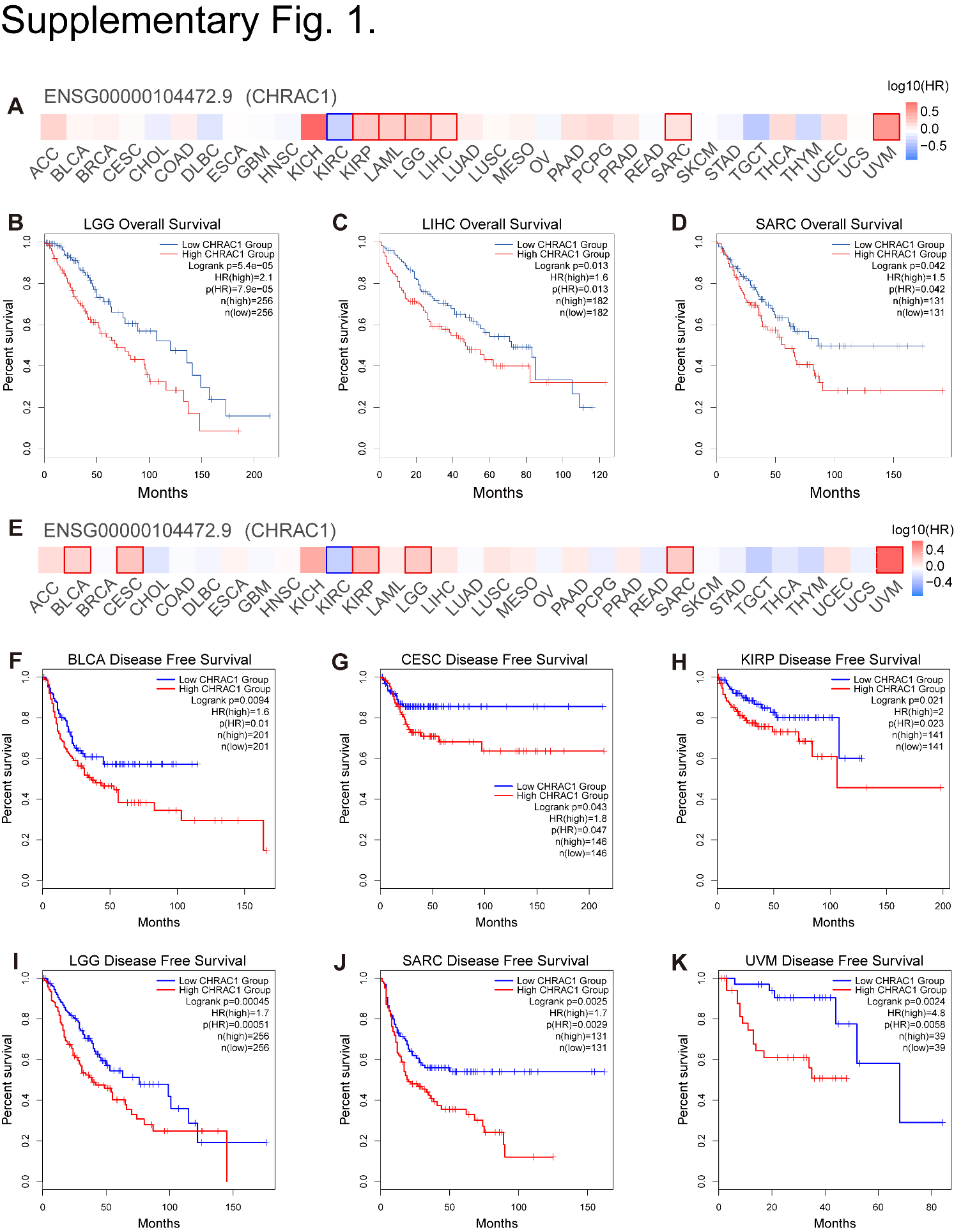
. Supplementary Figures

**Supplementary Fig. 1. High CHRAC1 expression is associated with overall survival and disease-free survival in a variety of cancer types.** (A) GEPIA2 was used to build an overall survival map of CHRAC1 in various cancer types. The survival maps with significant results are displayed as red boxes. (B–D) Kaplan–Meier curves depicting overall survival of two groups of patients with high or low CHRAC1 expression in LGG (B), LIHC (C) and SARC (D) cancer tissues (obtained from GEPIA2). The Kaplan–Meier plots with significant results are displayed. (E) GEPIA2 was used to build a disease-free survival map of CHRAC1 in various cancer types. The survival map with significant results were displayed as red box. (F–K) Kaplan–Meier curves depicting disease-free survival of two groups of patients with high or low CHRAC1 expression in BLCA (F), CESC(G), KIRP (H), LGG (I), SARC (J) and UVM (K) cancer tissues (obtained from GEPIA2). The red and blue lines indicate for high and low CHRAC1 groups, respectively. Data were from TCGA database
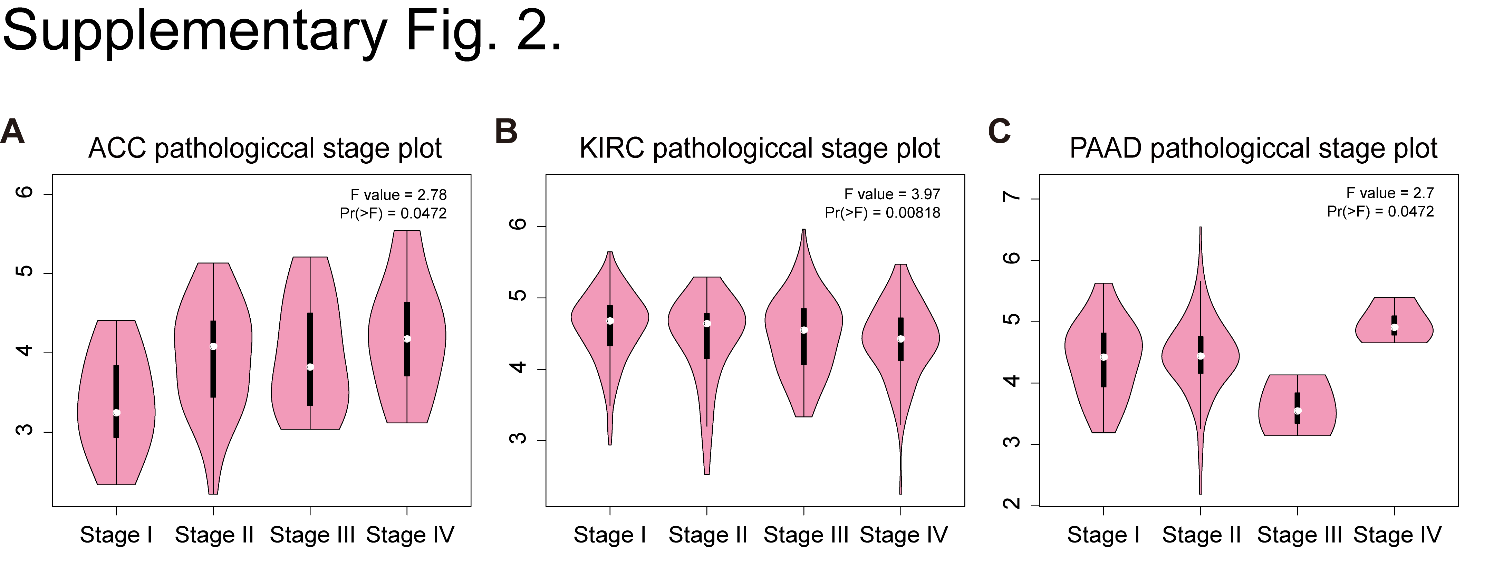
and were analyzed with GEPIA2 tool.

**Supplementary Fig. 2.** Correlation of CHRAC1 expression with main pathological stages (stage I, stage II, stage III, and stage IV) of ACC (A), KIRC (B) and PAAD (C) (Data are from TCGA database). Log2 (TPM + 1) was applied for log-scale.


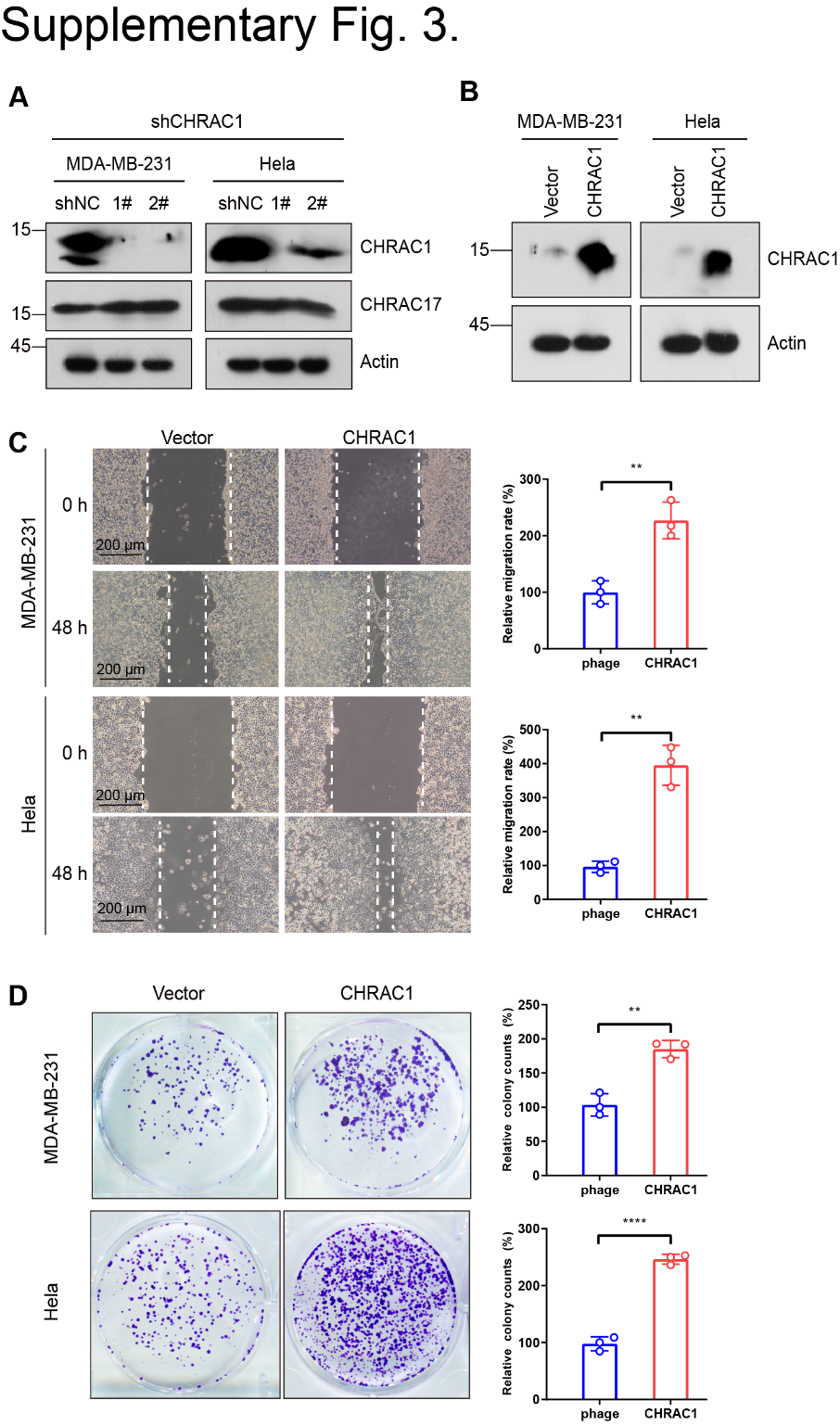


**Supplementary Fig. 3.** (A) Western blot analysis of protein expression of CHRAC1 and CHRAC17 in CHRAC1 knockdown MDA-MB-231 and Hela cells. (B) Overexpression of CHRAC1 in MDA-MB-231 and Hela cells detected by western blotting. (C) The effect of CHRAC1 overexpression on cell migration. The statistical analysis was demonstrated in histogram. (D) The effect of CHRAC1 overexpression on colony formation. The statistical analysis of clone numbers was demonstrated in histogram. ****P<0.0001, **P<0.01.

**2. RT-qPCR primers**

*CTGF*, 5′-AGGAGTGGGTGTGTGACGA-3′ and 5′-CCAGGCAGTTGGCTCTAATC-3′;

*CYR61*, 5′-CCTTGTGGACAGCCAGTGTA-3′ and 5′-ACTTGGGCCGGTATTTCTTC-3′;

*ANKRD1*, 5′-AGTAGAGGAACTGGTCACTGG-3′ and 5′-TGGGCTAGAAGTGTCTTCAGAT-3′;

*GAPDH*, 5′-GGAGCGAGATCCCTCCAAAAT-3′ and 5′-GGCTGTTGTCATACTTCTCATGG-3′;

*CHRAC1*, 5′-CGAGGTGTCCAGCATCAAC-3′ and 5′-CCTTTCCACTGCCGTGTCT-3′.
