# Supplementary figures and images for: Chromatin accessibility complex subunit 1 enhances tumor growth by regulating the oncogenic transcription of YAP in breast and cervical cancer
Source: PeerJ. 2024 Jan 10;12:e16752. doi: 10.7717/peerj.16752 (PMC10787542; doi:10.7717/peerj.16752)

**
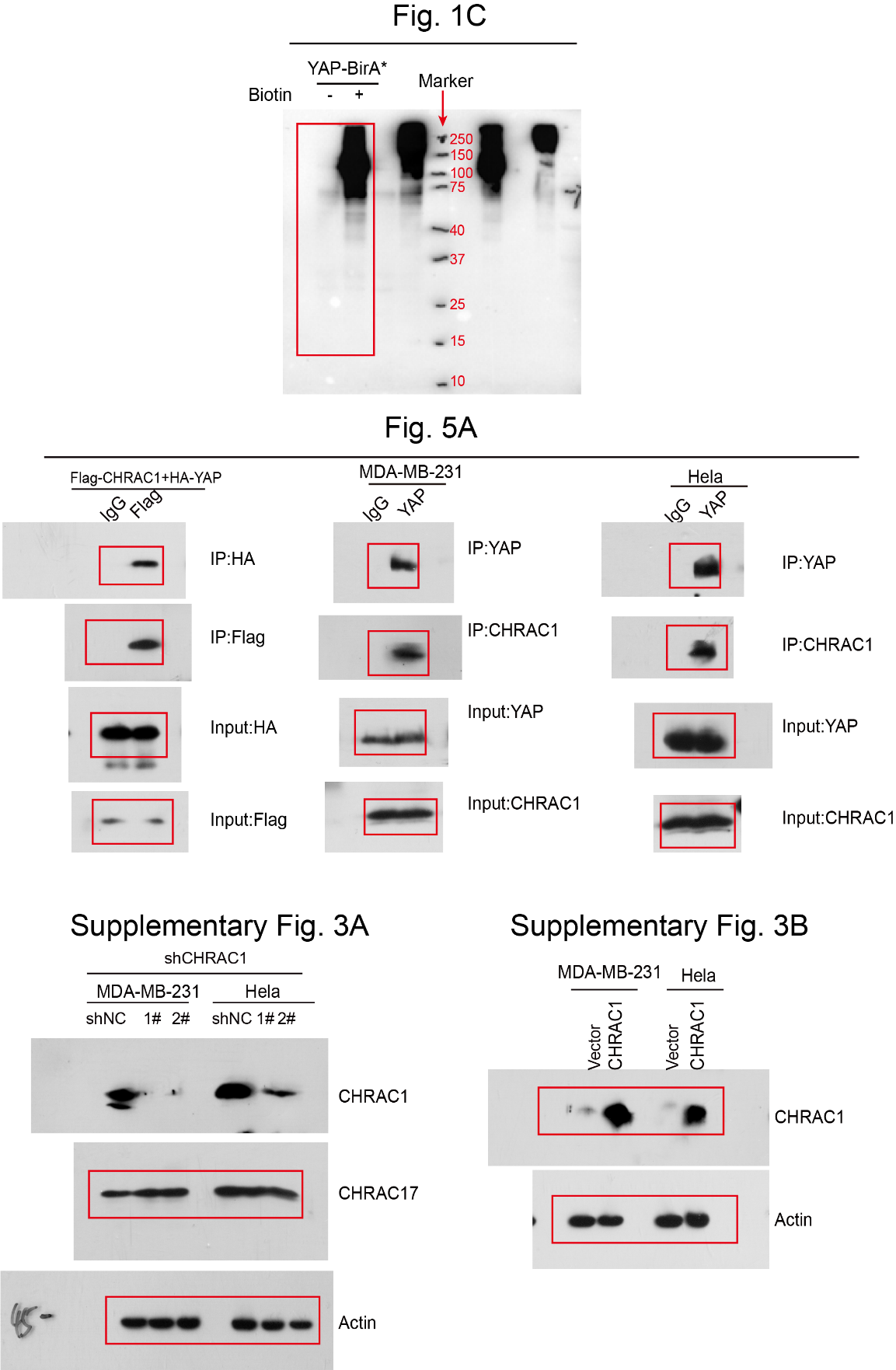
 Original western blot data**

Supplement: Supplemental Information 3 [file peerj-12-16752-s003.docx]
